# Socioeconomic status and exposure to disinfection by-products in drinking water in Spain

**DOI:** 10.1186/1476-069X-10-18

**Published:** 2011-03-16

**Authors:** Gemma Castaño-Vinyals, Kenneth P Cantor, Cristina M Villanueva, Adonina Tardon, Reina Garcia-Closas, Consol Serra, Alfredo Carrato, Núria Malats, Nathaniel Rothman, Debra Silverman, Manolis Kogevinas

**Affiliations:** 1 Centre for Research in Environmental Epidemiology (CREAL), Barcelona, Spain; 2 Municipal Institute of Medical Research (IMIM-Hospital del Mar), Barcelona, Spain; 3 CIBER Epidemiología y Salud Pública (CIBERESP), Barcelona, Spain; 4 Division of Cancer Epidemiology and Genetics, National Cancer Institute, Department of Health and Human Services, Bethesda, USA; 5 Universidad de Oviedo, Oviedo, Spain; 6 Unidad de Investigación, Hospital Universitario de Canarias, La Laguna, Tenerife, Spain; 7 Universitat Pompeu Fabra, Barcelona, Spain; 8 Consorci Hospitalari Parc Taulí, Sabadell, Spain; 9 Hospital General de Elche, Elche, Spain; 10 Centro Nacional de Investigaciones Oncológicas (CNIO), Madrid, Spain; 11 Department of Social Medicine, Medical School, University of Crete, Herakleion, Crete, Greece

## Abstract

**Background:**

Disinfection by-products in drinking water are chemical contaminants that have been associated with cancer and other adverse effects. Exposure occurs from consumption of tap water, inhalation and dermal absorption.

**Methods:**

We determined the relationship between socioeconomic status and exposure to disinfection by-products in 1271 controls from a multicentric bladder cancer case-control study in Spain. Information on lifetime drinking water sources, swimming pool attendance, showering-bathing practices, and socioeconomic status (education, income) was collected through personal interviews.

**Results:**

The most highly educated subjects consumed less tap water (57%) and more bottled water (33%) than illiterate subjects (69% and 17% respectively, p-value = 0.003). These differences became wider in recent time periods. The time spent bathing or showering was positively correlated with attained educational level (p < 0.001). Swimming pool attendance was more frequent among highly educated subjects compared to the illiterate (odds ratio = 3.4; 95% confidence interval 1.6-7.3).

**Conclusions:**

The most highly educated subjects were less exposed to chlorination by-products through ingestion but more exposed through dermal contact and inhalation in pools and showers/baths. Health risk perceptions and economic capacity may affect patterns of water consumption that can result in differences in exposure to water contaminants.

## Background

Environmental inequity has been defined as the disproportionate higher risk of environmental pollution exposure that some individuals suffer due to their race, age, ethnicity, or lower income [1]. It also refers to the equal treatment that all people should receive independently of their individual or social condition resulting from environmental policies, regulations and statutes [2]. Environmental inequity implies a wide variety of concepts, such as environmental classism, environmental justice or environmental racism [3].

Some individuals are more likely to be exposed to pollution due to low socioeconomic status. For instance, in the US, minority communities like African-American, Hispanic, Asian and Native American, are more exposed to air, water and soil pollutants released from hazardous waste sites [4]. Also, disadvantaged populations in terms of poverty, age or ethnicity live closer to industrial sites, being exposed to higher levels of airborne pollutants [5-7]. Biological or chemical pollution in drinking water is also a major concern for public health. Disinfection by-products (DBPs), inadvertently produced when drinking water is chlorinated, have been associated with adverse reproductive effects [8,9] and cancer [10-12]. Trihalomethanes (THM), the most prevalent component of the disinfection by-product mixture, are highly volatile and skin permeable. Consequently, exposure may occur through ingestion of water, inhalation and dermal contact while showering, bathing and swimming in pools. The higher molecular weight compounds in the mixture enter the body primarily through ingestion.

Our study aimed to evaluate the relation between DBP exposures and socioeconomic status (SES), under the hypothesis that the higher social classes would be less exposed to disinfection by-products through ingestion because of enhanced ability to purchase bottled water. Other routes of exposure, such as dermal absorption and inhalation during showering, bathing, and use of swimming pools, were also considered.

## Methods

### Study design and subjects

One thousand two-hundred nineteen (1,219) bladder cancer cases and one thousand two-hundred and seventy-one (1,271) hospital controls were recruited between 1998 and 2001 for the Spanish Bladder Cancer case-control study from 18 hospitals in 5 regions of Spain: Barcelona, Vallès/Bages, Alicante, Tenerife and Asturias. Subjects were 21-80 years old [11]. In the present study, only controls were included in the analysis. Controls were selected from patients admitted to the participating hospitals mainly for minor surgery or trauma. The main diagnoses in hospital admissions were hernias (37%), other abdominal surgery (11%), fractures (23%), other orthopedic conditions (7%), hydrocele (12%), circulatory conditions (4%), dermatological conditions (2%) and ophthalmologic conditions (1%). Eighty-eight percent (88%) of the controls completed a face-to-face computer-assisted personal interview (CAPI) that included socio-demographic information, smoking, occupational history, lifetime residential history, environmental exposures, medication, and family history of cancer. The study was approved by the Ethics Committees of all participating institutes; a written informed consent was obtained from all patients.

### Chlorination by-products exposure

Lifetime residential history was collected from subjects for each place of residence of longer than one year duration. Information requested included the main type of water consumed at each residence (i.e., public water supply, private well or bottled water), although it did not request information about changes of type of drinking water in the same residence. Other water ingestion information included: average daily water consumption (including water-based fluids such as coffee and tea); average frequency and duration of showers and/or baths; and ever lifetime swimming pool attendance. Data on current and historical levels of THM were collected from water utilities. In addition, a central laboratory measured THM in 113 tap water samples. Stratifying the regions according to the levels of THM measured, Barcelona, Vallès/Bages and Alicante would be included in the high THM levels area (mean 64, SD 27 μg/l) while Asturias and Tenerife have lower THM levels (mean 17, SD 13 μg/l) [13]. Lifetime individual exposure indices were calculated, as described elsewhere [14], merging individual and municipal databases by year and municipality, obtaining individual year-by-year average THM levels. Several individual exposure indices were created: current residential THM level, as the dichotomous variable (below or above the median - 48 μg/l) level of THM in the residence at the time of interview; average residential THM exposure, i.e. time-weighted average municipal THM level (μg/l) for all residences over age 15, as a dicthomous variable of levels below or above the median (26 μg/l); swimming pool attendance, as a dichotomous variable: attending a swimming pool once or more than once per year contrasted with never (or less than once per year); showers and baths, as a dichotomous variable below or equal to the median of minutes/day spent in the bath/shower (≤7 min/day) or above the median. The reproducibility of the questions about showering, bathing and swimming was evaluated in a subsample of the study subjects, obtaining more than 90% agreement in the answers [10].

### Socio-economic status

Educational level achieved by subjects was classified in 4 categories: illiterate, incomplete primary school, complete primary school (education through 13-14 years of age), and high school (through 17-18 years), or higher education. For some analyses these were grouped in two categories: low education (subjects with primary school or lower) and high education (subjects with higher than primary education). Household income at the time of the interview was recorded as a categorical variable, as was the number of family members living on that income. The variables were then combined into income per person, with 3 categories: low (<300 Euros/month), medium (300-600 Euros/month) and high income (>600 Euros/month). The response rate for this variable was lower (75%) than for the other variables (99%).

### Statistical analysis

We cross-tabulated social class and type of water variables, and used the chi-square test. Logistic regression was used to estimate the odds ratios (OR) and 95% confidence intervals (CI) for the type of water consumed by socioeconomic status adjusting for smoking status (pack-years), age, gender, area (5 groups: Barcelona, Vallès/Bages, Alicante, Tenerife and Asturias) and average municipal THM level for all residences over age 15. The analyses of THM and water source were restricted to subjects with exposure information for at least 70% of the exposure window examined (from 15 years of age until disease or interview) [8]. The analysis was performed using Stata, release 8.2 (StataCorp, 2005, College Station, TX, USA).

## Results

Characteristics of study subjects are shown in Table 1. The population includes 71% of ever smokers and 48% of people with less than primary school education. Educational level achieved differed between regions. The proportion of subjects with completed high school or higher education was 23% in Barcelona, 20% in Vallès/Bages, 18% in Alicante, 15% in Asturias and 12% in Tenerife.

**Table 1:** Main characteristics of the population

		n	%
Gender	Male	1105	87%

	Female	166	13%

Age; mean (sd)		65 (10)	

			

Region	Barcelona	247	19%

	Vallès/Bages	190	15%

	Alicante	84	7%

	Tenerife	226	18%

	Asturias	524	41%

			

Smoking status	Never smokers	367	29%

	Occasional smokers	97	8%

	Former smokers	510	40%

	Current smokers	291	23%

			

Education	Illiterate	368	30%

	Primary school incomplete	224	18%

	Primary school complete	443	36%

	High school/higher education	212	17%

			

Income per person	Low, <300 euros/month	308	32%

	Medium, 300-600 euros/month	464	47%

	High, >600 euros/month	176	19%

Note: some of the variables have missing values: smoking status (n = 6), education (n = 24), income per person (n = 323).

More than 60% of the population ever drank water from a public water supply and 13% had attended swimming pools (Table 2). A public water supply was the main source of drinking water at the time of the interview for 71% of subjects in Barcelona, 66% in Asturias, 60% in Tenerife, 55% in Vallès/Bages, and 46% in Alicante. There are significant differences in the use of bottled water by area of residence and the level of disinfection by-products. Among subjects living in high THM regions, the percentage that consumed bottled water was between 27 and 41% whereas 15 to 24% of subjects in low THM level areas drank bottled water.

**Table 2:** Percentages of drinking water type consumed at home, use of pools and duration of bath/shower by educational level

	Illiterate		Primary school incomplete		Primary school complete		High school or higher education		p-value	TOTAL	
	**n**	**%**	**n**	**%**	**n**	**%**	**n**	**%**		**n**	**%**

Source of drinking water, currently											

Tap water	243	69%	131	61%	265	62%	116	57%		755	63%

Bottled	59	17%	54	25%	99	23%	67	33%		160	23%

Springs and wells	48	14%	29	14%	61	14%	22	11%	0.003	279	14%

											

Current average residential THM level (μg/l)											

Low (<= 48 μg/l)	103	48%	69	46%	155	54%	59	38%		386	48%

High (>48 μg/l)	111	52%	80	54%	133	46%	97	62%	0.014	421	52%

											

Source of drinking water at the longest residence											

Tap water	227	63%	126	57%	258	58%	136	64%		747	61%

Bottled	39	11%	40	18%	67	16%	44	21%		190	15%

Springs and wells	96	26%	54	25%	116	26%	32	15%	0.002	298	24%

											

Lifetime average residential THM level, from age 15 (μg/l)											

Low (<= 26 μg/l)	132	59%	62	47%	155	54%	59	39%		408	51%

High (<26 μg/l)	93	41%	71	53%	133	46%	91	61%	0.002	388	49%

											

Ever swimming pool attendance											

No	259	93%	162	89%	307	88%	134	74%		862	87%

Yes	21	7%	21	11%	41	12%	46	26%	<0.001	129	13%

											

Showers/baths											

Short (<= 7 min/day)	150	60%	87	52%	179	55%	77	44%		493	54%

Long (>7 ming/day)	99	40%	81	48%	149	45%	98	56%	0.010	427	46%

Note: the sum of the columns for the different variables may not be equal due to missing values

Bottled water was the main source of drinking water at home at the time of interview among 17% of illiterate subjects and 33% of those with a high school degree or higher education, while the corresponding proportions for public water supply use were 69% and 57% (Table 2). There was also a difference in bottled water consumption at home when considering the longest residence from 11% of illiterate subjects to 21% of highly educated subjects, although we did not observe inverse rates of use of tap water. The most highly educated subjects lived in residences with, on average, higher THM levels (36 μg/l) than illiterate subjects (29 μg/l, Table 2), although no clear pattern of increasing THM levels with educational level was seen. In the multivariate analyses, considering the use of public supply water among the illiterate as the referent, subjects who had attended primary school had a two-fold probability of consumption of bottled drinking water (OR = 2.1; 1.2-3.7) while the more highly educated subjects had over three times the probability of consuming bottled water (OR = 3.3; 1.8-6.0).

The more highly educated subjects tended to take longer baths or showers and attended swimming pools more than persons with a lower educational level (Table 2). Subjects with high school education have a three fold higher probability (OR = 2.7, 95%CI 1.6-4.6) of taking bath/showers longer than 7 min/day than illiterate subjects and a 1.6 (95%CI 1.0-2.6) higher probability than subjects with primary school education. Use of swimming pools (ever/never) was also more likely in subjects with a high school education or above compared to the illiterate (OR = 3.4, 95%CI 1.6-7.3) while smaller differences were observed for subjects having attended (OR = 1.3, 95%CI 0.5-3.0) or completed (OR = 1.6, 95%CI 0.7-3.5) primary school.

Income was also used as a measure of socio-economic status, showing trends similar to those observed for educational level. Twenty-nine percent (29%) of subjects in the high-income category drank bottled water compared with 18% in the low-income category (p = 0.047). Eight percent of the low-income subjects were swimming pools attendees, in contrast to 21% of those with higher income (p < 0.001). The time spent in the shower and bath per day did not show a statistically significant difference by income (p > 0.05).

**Figure 1:**
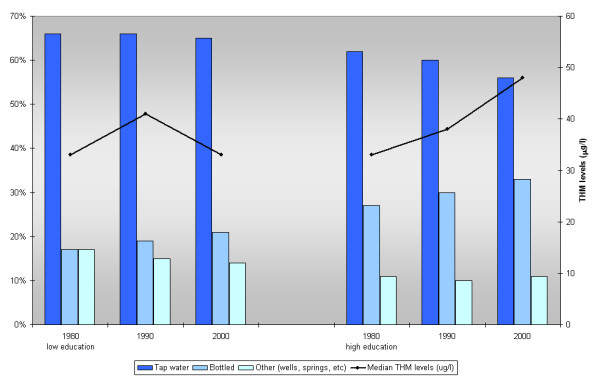
**Type of water consumed and THM levels in 3 time periods by educational status**.

An increase in the consumption of bottled water from 1980 to 2000 was observed overall, from 18% in 1980 to 20% in 1990 and 23% in the year 2000. When stratifying by socioeconomic categories as defined by education (Figure 1), among subjects with lower levels of education, there were no changes over time in the use of water from public supplies (66% during 1980-1990 and 65% during 2000). A decrease from 63% to 56% was observed for the same time period among subjects with higher education. In the same period there was an increase of bottled water use from 17% to 21% among subjects with a lower educational level and from 26% to 33% among the more highly educated subjects, along with an increase in the levels of THMs, only observed in the latest category of education (Figure 1).

The trend of water consumption at work according to educational level was similar to that observed in the home, along with a decrease in water consumption from public supplies.

## Discussion

The main source of drinking water in the Spanish population is from public water supplies. In our study population, the use of bottled water increased from 18% to 23% over the 20 year period from 1980 to 2000, with the greatest increase occurring where THM levels were the highest. The use of bottled water as the main source of drinking water, the use of swimming pools and the frequency and duration of showers and baths was higher among subjects with higher education and income compared to those with lower levels.

The higher proportion of bottled water use among the higher social classes could be explained by the higher income available to spend on purchase of bottled water, related to taste, perception of quality or perception of risk. However, we did not have information about the reasons that led people to switch from tap or other sources to bottled water. A more general perception of risk related to exposure to disinfection by-products has only recently been publicized in Spain and changes related to use of bottled water in the 1980 s or 1990 s are probably not due to health risk perception. This perception, if existed, would affect the type of drinking water consumed, but not other water uses that could lead to exposure to DBPs through inhalation or dermal absorption.

The association between socio-economic status, environmental exposures and health effects is not always obvious. For instance, allergies and hay fever have an inverse relation with social class, with lower prevalence in lower socioeconomic classes [15]. In the case of DBP, this relation is complex and can vary regionally. In our Spanish study population, subjects with a higher socioeconomic status tended to live in areas with elevated DBP, had a lower exposure from ingestion (because of elevated consumption of bottled water) and a higher exposure from inhalation and dermal absorption (due to the more frequent use of pools and longer showers and baths).

A concern in these analyses is the measurement of social class. We used two measures of socioeconomic status (education and income) and results were similar for both indices, although the percentage of missing data for the income variable was high. The most important and frequently used indicators of socioeconomic status are education, income and occupation [16]. There is continuing debate regarding the best measure, and whether these measures should be used separately or as a composite index [15]. Socioeconomic indices measure different elements in social position and cannot be treated equally as measures of social class [17]. Education has been extensively used as an indicator of social class, as it is stable over time. In our study population, a low percentage of participants reached secondary or higher education (17%), which reflects the percentage in the general population of Spain between 55-64 years old (16%) [18].A potential limitation is that education may not capture changes in social class that occur in adulthood [19].

## Conclusions

In conclusion, in this population the use of bottled water as a source of drinking water, the use of swimming pools, and the frequency and duration of showers and baths was higher among subjects with higher education and income compared to those with lower levels of either. This would result in lower exposure to chlorination by-products through ingestion among subjects of higher socioeconomic status but higher exposure through dermal contact and inhalation. Health risk perceptions and economic capacity may affect patterns of water consumption that, however, may not necessarily result in differences in exposure to water contaminants. Our findings, of course, are specific to the study population of the five regions of Spain, formed mainly by elderly males. The broader implication of this analysis is that the relationship between socioeconomic status and exposure to DBP is complex and is dependent on the social and environmental context of exposed populations.

## List of abbreviations

DBP(s): Disinfection By-product(s); THM(s): Trihalomethane(s); SES: Socioeconomic Status; CAPI: Computer-Assisted Personal Interview; OR: Odds Ratio; CI: Confidence Interval.

## Competing interests

The authors declare that they have no competing interests.

## Authors' contributions

GC performed the statistical analysis and drafted the manuscript with input from all investigators. KPC participated in the study design, statistical analysis and helped to draft the manuscript. CMV participated in the study design and in the analysis and interpretation of data. AT participated in the study design and acquisition of data. RG participated in the study design and acquisition of data. CS participated in the study design and acquisition of data. AC participated in the study design and acquisition of data. NM participated in the study design and acquisition of data. NR and DS participated in the study design and enrollment of patients. MK participated in the study design, enrollment of patients, statistical analysis and helped to draft the manuscript. All authors read and approved the final manuscript.

## Authors' information

The participating study centres in Spain were: Institut Municipal d'Investigació Mèdica, Universitat Pompeu Fabra, Barcelona--Coordinating Center (M Kogevinas, N Malats, F X Real, M Sala, G Castaño, M Torà, D Puente, C Villanueva, C Murta, J Fortuny, E López, S Hernández, R Jaramillo); Hospital del Mar, Universitat Autònoma de Barcelona, Barcelona (J Lloreta, S Serrano, L Ferrer, A Gelabert, J Carles, O Bielsa, K Villadiego); Hospital Germans Tries I Pujol, Badalona, Barcelona (L Cecchini, J M Saladié, L Ibarz); Hospital de

Sant Boi, Sant Boi, Barcelona (M Céspedes); Centre Hospitalari Parc Taulí, Sabadell, Barcelona (C Serra, D García, J Pujadas, R Hernando, A Cabezuelo, C Abad, A Prera, J Prat); ALTHAIA, Manresa, Barcelona (M Domènech, J Badal, J Malet); Hospital Universitario, La Laguna, Tenerife (R García-Closas, J Rodríguez de Vera, A I Martín); Hospital La Candelaria, Santa Cruz, Tenerife (J Taño, F Cáceres); Hospital General Universitario de Elche, Universidad Miguel Hernández, Elche, Alicante

(A Carrato, F García-López, M Ull, A Teruel, E Andrada, A Bustos, A Castillejo, J L Soto); Universidad de Oviedo, Oviedo, Asturias (A Tardón); Hospital San Agustín, Avilés, Asturias (J L Guate, J M Lanzas, J Velasco); Hospital Central Covadonga, Oviedo, Asturias (J M Fernández, J J Rodríguez, A Herrero); Hospital Central General, Oviedo, Asturias (R Abascal, C Manzano, T Miralles); Hospital de Cabueñes, Gijón, Asturias (M Rivas, M Arguelles); Hospital de Jove, Gijón, Asturias

(M Díaz, J Sánchez, O González); Hospital de Cruz Roja, Gijón, Asturias (A Mateos, V Frade); Hospital Alvarez-Buylla, Mieres, Asturias (P Muntañola, C Pravia); Hospital Jarrio, Coaña, Asturias (A M Huescar, F Huergo); Hospital Carmen y Severo Ochoa, Cangas, Asturias (J Mosquera).
